# The globally invasive small Indian mongoose *Urva auropunctata* is likely to spread with climate change

**DOI:** 10.1038/s41598-020-64502-6

**Published:** 2020-05-04

**Authors:** Vivien Louppe, Boris Leroy, Anthony Herrel, Géraldine Veron

**Affiliations:** 1 Institut de Systématique, Evolution, Biodiversité (ISYEB), Muséum national d’Histoire naturelle, CNRS, Sorbonne Université, EPHE, Université des Antilles, 57 rue Cuvier, CP 51, 75231 Paris, Cedex 5 France; 2 Unité Biologie des Organismes et Ecosystèmes Aquatiques (BOREA UMR 7208), Muséum National d’Histoire Naturelle, Sorbonne Universités, Université de Caen Normandie, Université des Antilles, CNRS, IRD, Paris, France; 3 Département Adaptations du Vivant (FUNEVOL, UMR 7179), Muséum National d’Histoire Naturelle, CNRS, Paris, France

**Keywords:** Ecological modelling, Climate-change ecology, Invasive species

## Abstract

Invasive alien species represent one of the major factors of global loss of biodiversity and disruption of natural ecosystems. The small Indian mongoose, *Urva auropunctata*, is considered one of the wild carnivore species with the greatest negative impact on global biodiversity. Understanding of the factors underpinning the species’ distribution and potential dispersion in a context of climate change thus appears crucial in the conservation of native ecosystems. Here we modelled the current and future climatically favourable areas for the small Indian mongoose using Ecological Niche Modelling based on data sets filtrated in environmental spaces. Projections from these models show extensive current favourable geographical areas, covering continental and insular regions within tropical and sub-tropical latitudes. Moreover, predictions for 2050 reveal that climate change is likely to expand current favourable areas north of the current favourable spaces, particularly in Eastern Europe. This climate-induced expansion is particularly worrisome given that the species is already spreading in the Balkan region. Our projections suggest that it is very likely that the small Indian mongoose will have an increasing influence on ecosystems and biodiversity in Europe by 2050.

## Introduction

The introduction of species outside of their native range in ecosystems in which they are competitive and where perennial populations can develop represents one of the major causes of biodiversity decline^1^, as well as a potential threat to human health and economy^2^. Understanding and identifying the determinant factors in the establishment and development of introduced populations is a major challenge in the effort to protect and conserve global biodiversity. By assessing present and future risks through different scenarios of environmental change, Ecological Niche Modelling (ENM) represents an important tool in the design and implementation of ecosystem management plans in the context of climate change^3–7^.

The small Indian mongoose, *Urva auropunctata*, is a small carnivore whose native distribution extends from Iraq to Myanmar, covering Iran, Pakistan, Northern India, Nepal and Bangladesh^8^. However, the species was introduced to a large number of regions around the globe between the late 19th and early 20th century: in the Caribbean from 1870-72, in Hawaii in 1882, in Fiji in 1883, in the South American continent and in several islands of the Mascarenes from 1900, and finally on several Japanese and Croatian islands from 1910. These introductions were primarily conducted for the purpose of biological control in order to limit the proliferation of rats in sugar cane plantations and to eradicate populations of venomous snakes (for instance *Bothrops lanceolatus* in Martinique, *Trimeresurus flavoviridis* in the Japanese archipelago, or *Vipera ammodytes* on several islands of the Adriatic Sea^9^). Today, the species is present in more than sixty islands around the globe^10^, and populations are spreading on the European continent in Croatia, Bosnia-Herzegovina and Montenegro^11,12^. The species’ current distribution attests to its ability to occupy a wide range of habitats, including open landscapes, scrubs, dry and temperate forest environments, but also humid forest, mangroves and coastal habitats^13,14^. The small Indian mongoose is also present in highly anthropized environments such as farmland, peri-urban and urban areas^15^. Finally, the species can be observed at high altitude, up to 2100 meters in its native range^16^, and has been observed up to 3000 meters in some invaded areas^15^. The small Indian mongoose has an opportunistic although mainly carnivorous diet and feeds on small vertebrates (reptiles, birds, mammals), but also bird and reptile eggs, crustaceans, insects, seeds and other vegetable items, or human waste^9,17^. The negative impact of the small Indian mongoose on native species in introduced regions was observed soon after the first introductions during the late 19th century^18–21^. Even more so the introduction of the small Indian mongoose has been strongly correlated with the decline, extirpation and extinction of many species of amphibians, reptiles, birds, and mammals^9,22^. In addition, the small Indian mongoose may also present a health risk, the species being a vector of pathologies such as rabies^23–25^, leptospirosis^26–28^, salmonellosis^29^, or bartonellosis^30^, and a potential reservoir for parasites^31^. Consequently, the small Indian mongoose is now considered as one of the introduced wild carnivores with the biggest impact globally^32,33^, demonstrating the need for a better understanding of the mechanisms underpinning its distribution and potential dispersal in a context of climate change.

By associating geo-referenced occurrences and environmental variables, ENM appears as one of the most effective tools to assess and quantify species-environment relationships, allowing the modelling of environmental envelopes (or niches), and the identification of geographical areas favourable to a species. ENM have thus been widely used in a large number of disciplines, such as conservation biology (e.g.^34–36^), parasitology (e.g.^37,38^), and paleontology (e.g.^39,40^). The use of ENMs in a growing number of studies has greatly contributed to their development, and also highlighted various constraints and difficulties inherent to this technique^41–44^. One of these difficulties lies in the fact that early approaches relied on the principle of niche conservatism, which assumes the retention of inherited niche-related ecological traits over time and space^45^. However this assumption has been particularly challenged by frequent occurrence of niche shifts in plants (e.g.^46,47^), insects^48,49^, amphibians^50,51^, reptiles^52^ and birds^53^. Considering niche conservatism or not is particularly important regarding the selection of data used in modelling, especially in invasive species studies. Indeed, if niche conservatism is considered, only native occurrences are used in models predicting areas favourable for a species. Conversely, if niche conservatism is not considered, modelling the niche of an introduced species requires model calibration using both native and non-native range occurrences^54–56^. In consequence, evaluation of niche conservatism represents a crucial step in invasive species niche modelling^56,57^.

Bellard *et al*.^58^ investigated the areas favourable to the small Indian mongoose through an ENM analysis of the «100 world’s worst invasive species» from the list of the Invasive Species Specialist Group^59^. However, the conclusions of this work regarding the small Indian mongoose appear limited by the large number of taxa included in the final projections and by a taxonomic bias, using data of both *Urva auropunctata* and *Urva javanica*, as the two species were considered conspecific until recently, under the name *Herpestes javanicus*^60–62^. Bellard *et al*.^58^ conclusions regarding the small Indian mongoose also appear limited by sampling biases related to the absence of filtering of occurrence data. Such sampling biases may alter the results by incorrectly assessing the significance of environmental variables in oversampled regions^63^, resulting in overfitting of models^64^ and artificial inflation of model accuracy in these areas^65,66^. To overcome this bias, different methods of filtering of occurrence data have been proposed, such as filterings in geographical spaces, by the application of buffer distances for each occurrence^64^. However, methods relying on filtering in the environmental space occupied by the species have recently demonstrated significant improvement of model performance^67,68^, notably in the case of invasive species^69^.

Given the impact of the small Indian mongoose on native ecosystems in introduced areas, it is essential to identify the regions that may be at risk of colonization under climate change scenarios. The objective in this study was to model the current and future climatically favourable areas for the small Indian mongoose at a global scale using environmental niche models. To this end, we first compared the bioclimatic space occupied by the species in its native range versus the non-native areas. Secondly, we assessed the bioclimatic envelope of the small Indian mongoose using a modelling protocol based on an environmental filtering of species presence and the selection of pseudo-absences outside the environmental space occupied by the species. We applied an ensemble modelling procedure based on nine statistical models, and predictions for 2050 were realized using two future scenarios based on the extreme ends of the Representative greenhouse gas Concentration Pathways (RCP) scenarios, and a consensus of three Global Circulation Models (GCM).

## Methods

### Species occurrence data

**Figure 1:**
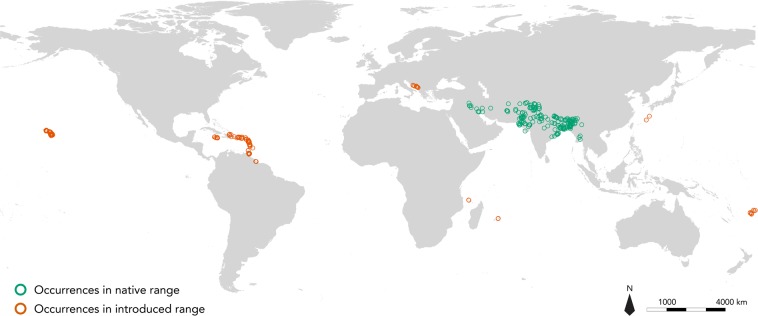
Occurrences of small Indian mongoose used in the present study. Green circles correspond to occurrences in the native range of the species. Orange circles correspond to occurrences in the introduced range of the species.

Occurrences of the small Indian mongoose *U. auropunctata* were compiled from the online databases VertNet and GBIF, in addition to the databases of the Guadeloupe National Park, Martinique Regional Park, Saint-Martin Natural Reserve, French national wildlife organizations, recent literature, museum specimens and personal observations. Only recent observations (from 1950 to the present day) with complete and precise locations were selected, giving a total of 971 occurrences (Table S1). All occurrence records were aggregated into 0.08° cells corresponding to the resolution of environmental variables, resulting in a total of 403 records (Fig. 1).

### Environmental data

Current and future favourable environmental envelopes for the small Indian mongoose were calculated using 19 bioclimatic variables (derived from temperature and precipitation measures), averaged for the period 1950–2000 from the WorldClim 1.4 database^70^. As is apparent from its current distribution and the known ecology of the species, the small Indian mongoose is able to cope with, and found in, a great variety of natural and anthropogenic habitats. As such, bioclimatic variables represent appropriate and reliable parameters to portrait the current and future areas favourable for the species at a global scale. A 5 arc-minute spatial resolution (approximately 9 kilometers at the equator) was selected for all bioclimatic variables.

Future favourable envelopes for the small Indian mongoose were modelled using climate projections from global climate models (GCM) based on the Coupled Model Inter comparison Project Phase 5 (CMIP5) averaged for the period 2041–2060. Three GCMs were used (CNRM-CM5; GISS-E2-R; MIROC-ESM-CHEM; Table 1), for two Representative greenhouse gas concentration pathways scenarios (RCP): the most optimistic RCP2.6 (with a radiative forcing of +2.6 W/m² for the period 2000–2100), and the most pessimistic RCP8.5 (with a radiative forcing of +8.5 W/m²).

**Table 1:** Codes and descriptions of the bioclimatic variables and General Circulation Models selected for use in our models.

	Code	Description
Bioclimatic variables	Bio 2	Mean diurnal range (mean of monthly (max temp - min temp))
	Bio 7	Temperature annual range
	Bio 10	Mean temperature of the warmest quarter
	Bio 13	Precipitation of the wettest month
	Bio 19	Precipitation of the coldest quarter
General Circulation Models	CNRM-CM5	Centre National de Recherches Météorologiques, France
	GISS-E2-R	NASA Godard Institute for Space Studies, U.S.A.
	MIROC-ESM-CHEM	Japan Agency for Marine-Earth Science and Technology;Atmosphere and Ocean Research Institute (The University of Tokyo);National Institute for Envionmental Studies, Japan

Collinearity and the influence of the 19 bioclimatic variables were tested, using a protocol adapted from Leroy *et al*. (2014) and Bellard *et al*. (2016) (Method S1; Fig. S1). This allowed for the identification of five non-collinear variables that significantly influenced the distribution of the small Indian mongoose (Table 1; Fig. S2).

### Analysis of niche conservatism

Following the methodology proposed byWarren, Glor and Turelli^71^, and further developed by Broennimann *et al*.^56^, we calculated niche overlap, equivalence, and similarity between the native range (226 occurrences) and the invaded range (177 occurrences). Niche overlap, equivalency and similarity indices were calculated using a PCA approach and calibrated with the five selected environmental variables for each area of interest.

Along with the niche overlap test, niche expansion and unfilling were also calculated. The niche expansion index corresponds to environmental conditions in the invaded area that are absent in the native area. Conversely, unfilling refers to environmental conditions in the native area that are absent in the invaded area. Both indices vary between 0 and 1.

All niche conservatism tests were performed using the package ecospat v2.1.1^72^ implemented in the R software^73^.

### Data preparation and modelling process

Data preparation and pseudo-absence selection were realised following the methodology developed and assessed by Louppe *et al*.^69^. The preparation of presence-only data for ENMs included two important steps. The first one consisted of filtering the presence data to reduce autocorrelation and sampling bias (e.g. refs. ^67,74^). The second one consisted of selecting pseudo-absence data outside the environmental space resulting from the set of occurrences (i.e. the environmental space occupied by the species) to calibrate models. This procedure was realized using the R package geometry v0.4.3^75^.

Nine different modelling techniques were calibrated and evaluated^76–79^: Artificial Neural Networks – ANN^80^; Classification Tree Analysis - CTA^81^, Flexible Discriminant Analyses – FDA^82^; Generalised Additive Models – GAM^83^; Generalised Boosted Models – GBM^84^; Generalised Linear Models – GLM^85^; Multivariate Adaptative Regression Splines – MARS^86^; MAXimum ENThropy – MAXENT^87^; Random Forests – RF^88^. For each set of presences/pseudo-absences, model calibration was performed with 70% of the data. The remaining 30% were used for model evaluation. Models were calibrated and evaluated three times per set of presences/pseudo-absences. Model calibrations were realized with the R package BIOMOD 2 v3.3-7^89^.

Because our procedure is a presence/pseudo-absence procedure, we did not calculate discrimination capacity metrics (e.g. the area under the receiver operating characteristic curve or the true skill statistics) because (1) these metrics are designed to be calculated on real absences, and (2) they are dependent on prevalence, which can lead to spurious evaluations of ENMs^90,91^. Rather, we evaluated our models with the Boyce index, specifically developed for such data^92–94^. The Boyce index assesses how much model predictions match the observed distribution of species occurrences. Values of the Boyce index vary between −1 and +1. Positive values indicate a model with predictions that are consistent with the distribution of occurrences in the evaluation dataset whereas negative values indicate a model with predictions that are not consistent with the distribution of occurrences. Boyce index values close to zero mean that the model is not different from a random model. Models with a mean Boyce index higher than 0.7 were selected (Fig. S3). Consequently, five models were kept for modelling: GLM, GBM, GAM, FDA and MARS. These analyses were performed using the R package ecospat v2.1.1^72^.

Forecasts of current and future favourable areas were obtained by the ensemble forecasting method. Current and future ensemble forecasts represent consensual projections of the five modelling techniques, obtained through averaged distributions of favourability scores^89,95,96^. To discriminate current favourable and non-favourable areas, and visualize shifts by 2050, continuous probability of favourability were transformed into binary projections. These binary projections were obtained using a probability threshold that maximized the Sørensen value^91^. Sørensen indices were calculated using 70% of occurrences as training data, and the remaining 30% as testing data.

## Results

### Environmental space comparisons

**Figure 2:**
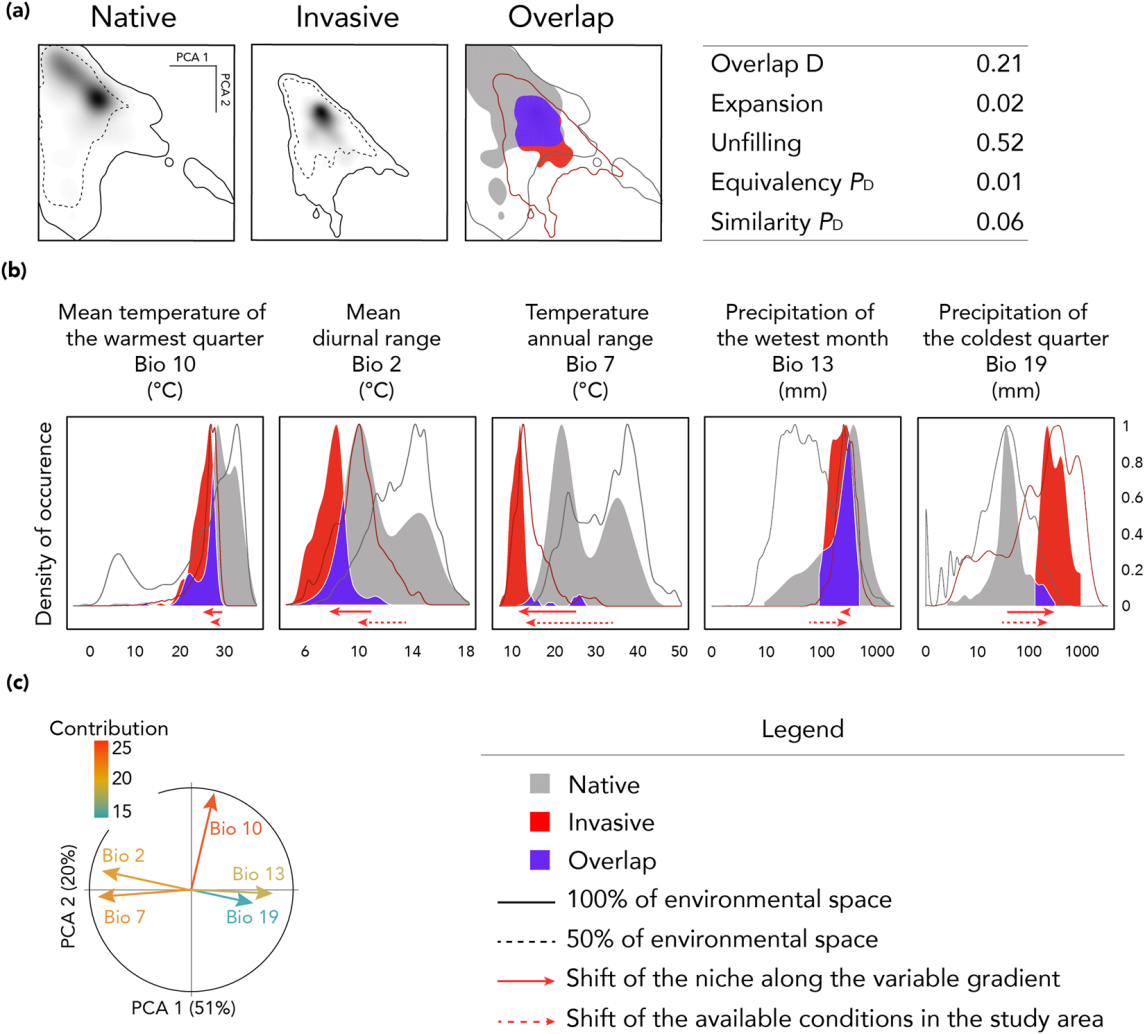
Analyses of environmental space shifts between the native range and the regions of introduction of the small Indian mongoose. (**a**) Environmental occupancy of *U. auropunctata* in native and non-native ranges along the two first axes of the PCA, and tests of niche overlap, niche equivalence and niche similarity between the native and the invaded areas niches. Grey to black gradients in native range graph represent the density of occurrence. Solid lines represent 100% of the available environmental space. Dashed lines represent 50% of the environmental space. (**b**) Occupancy of the niche along each variable gradient. A solid red arrow represents the shift of the non-native niche along the variable gradient. A dashed red arrow represents the shift of the available conditions in the non-native range. Solid lines represent 100% of available environmental space. Abscissa axes for precipitation variables are log-transformed to improve clarity. (**c**) Contribution of the bioclimatic variables to the two first axes of the PCA, and percentage of inertia explained by the two axes.

Our results indicated little overlap between the native niche and the niche of the invaded areas (D = 0.21; Fig. 2). Moreover, we found almost no niche expansion in invaded areas, but we found high niche unfilling, indicating that the species has not colonized all the possible environmental conditions shared with the native niche or that those conditions were absent from the non-native range (Fig. 2).

### Bioclimatic niche model

**Figure 3:**
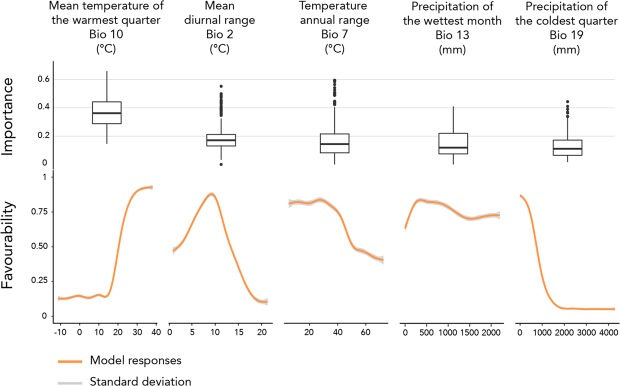
Variable importance and response curves of the favourability value predicted by the models. Dashed blue lines represent favourability threshold. Solid blue lines represent the favourable range along the variable gradient.

Model responses to bioclimatic variables appear sharper for the mean temperature of the warmest quarter (bio10), the mean diurnal range (bio2) and the precipitation of the coldest quarter (bio19; Fig. 3), indicating high favourability of temperatures of the warmest quarter above 20 °C, mean diurnal range between 0 and 14 °C, and precipitation during the coldest quarter below 900 mm. Responses for the temperature annual range (bio7) and the precipitation of the wettest month (bio13) appear less restrictive as almost all conditions are identified as favourable.

**Figure 4:**
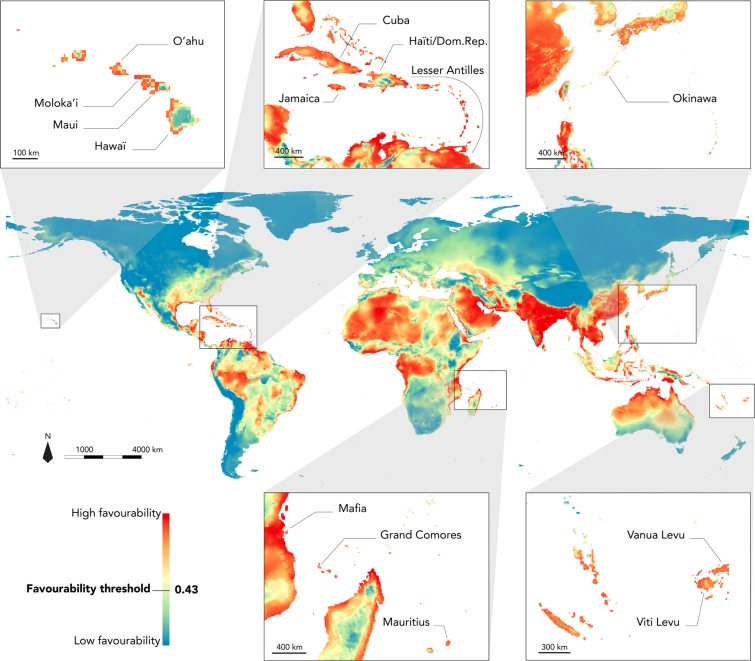
Projection of current global bioclimatic favourability for the small Indian mongoose with focus on regions where the species has been introduced. Locations where the species is currently present are indicated (Lesser Antilles: Puerto Rico, Vieques, St Thomas, Tortola, St Croix, St Martin, St Kitts, Nevis, Antigua, Guadeloupe, Fajou, La Désirade, Marie-Galante, Martinique, St Lucia, Barbados, St Vincent, Carriacou, Grenada, and Trinidad). Figure generated using the software R v3.5 (https://www.r-project.org) and QGIS v2.14.20 (https://qgis.org/).

Our model predicted favourable areas for the small Indian mongoose over most of the tropical and sub-tropical regions of the globe, covering 31% of all terrestrial areas, and encompassing most of the islands within these latitudes (Fig. 4; Figs. S4 and S5). As expected, islands near invaded regions in the Caribbean, the Mediterranean, and the Pacific, as well as the Japanese archipelago, appear highly favourable for the species.

**Figure 5:**
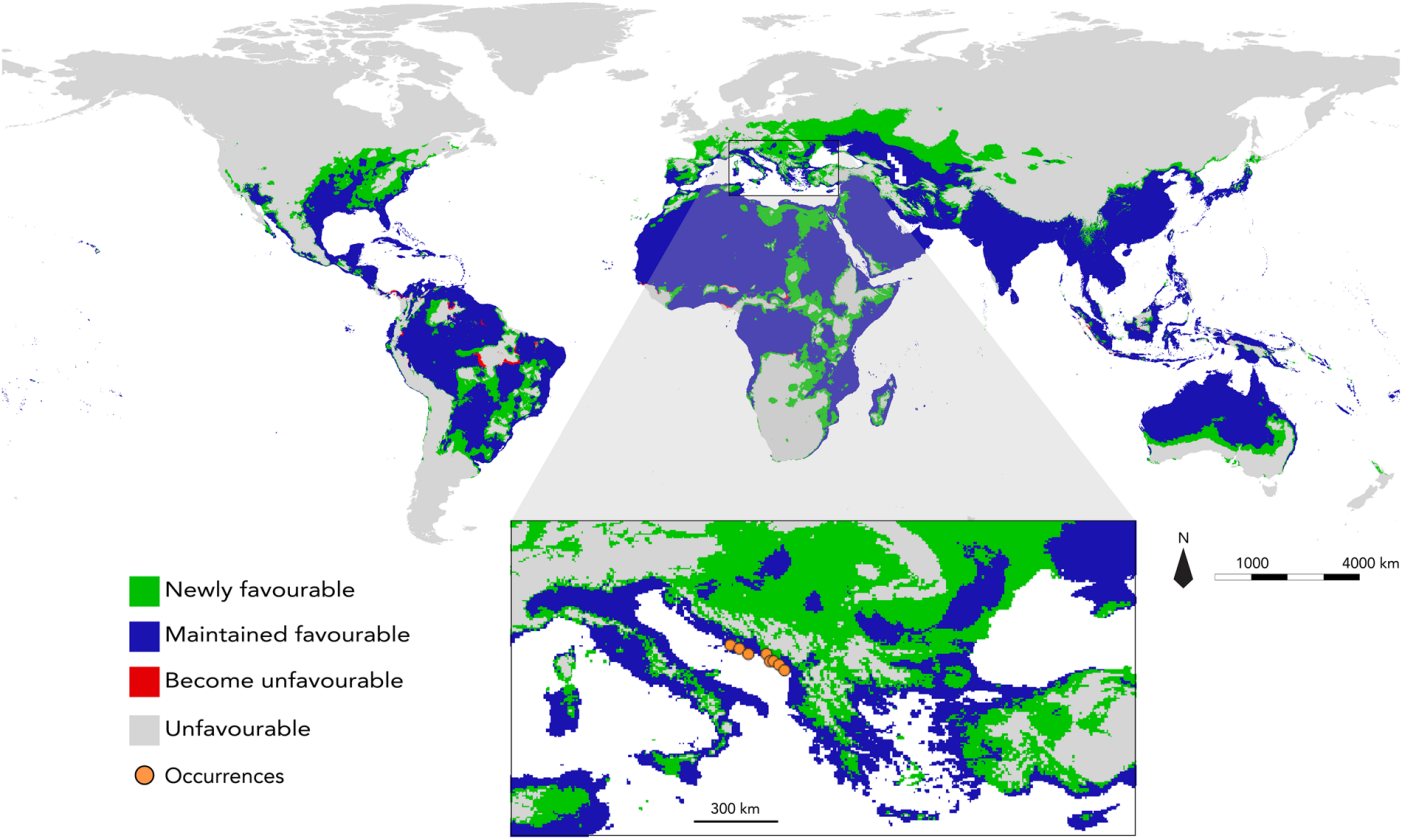
Projection of futur global bioclimatic favourability for the small Indian mongoose. Predicted favourable range change for *U. auropunctata* by 2050 according to scenario RCP8.5. Unfavourable: areas that are currently unfavourable and predicted to remain unfavourable in the future; Become unfavourable: areas currently favourable and predicted to become unfavourable in the future; Maintained favourable: areas that are currently favourable and predicted to stay favourable in the future; Newly favourable: areas that are currently unfavourable and which are predicted to become favourable in the future. Figure generated using the software R v3.5 (https://www.r-project.org) and QGIS v2.14.20 (https://qgis.org/).

By 2050, favourable areas are predicted to increase with both RCP scenarios, with an expansion of respectively 18% and 25% of the current favourable areas with RCP2.6 and RCP8.5 (Figs. 5; S6). Areas that will become unfavorable by 2050 are scarce and mainly located in the central part of South America and Africa. Conversely, model predicted broad newly favorable areas, particularly increasing from the northern border of the current favourable regions in Eastern Europe, the Caucasus and central Asia, as well as in North America. Results also show that currently occupied areas will be preserved in 2050.

## Discussion

This study presents the most complete analysis, to date, of the bioclimatic envelope favourable to the presence of one of the most invasive and devastating carnivores, the small Indian mongoose *Urva auropunctata*, and the first forecast of current and future areas favourable to the species at a global scale. Niche model was computed using occurrences sampled from both native and non-native areas. Our results attest of the tolerance of the species to high temperatures and a wide range of precipitation conditions. Consequently, current favourable areas extend broadly within tropical and sub-tropical latitudes. Moreover, climate change projections for 2050 predicted an expansion of current favourable areas, highlighting the potential risk for native ecosystems.

### Comparison of the bioclimatic space occupied by the species in its native range versus the non-native areas

Comparative analyses of the niches in the native and the invaded areas highlighted significant differences between both niches. These differences appear to be explained more by niche unfilling than by niche expansion of the niche in the invaded areas. Niche unfilling corresponds to the part of the available climatic conditions that is present in both native and invaded range, but which is occupied by the species only in the native range^47,97^. Unfilling is then commonly interpreted as the result of dispersal processes such as the impossibility to reach new areas, slow dispersal, or ongoing colonization. In the case of the small Indian mongoose, this unfilling value can be explained mostly by the introduction of the species mainly in insular environments, drastically limiting its dispersion. On the other hand, its introduction in mainland regions in Surinam and Guyana, as well as in Croatia, Bosnia and Herzegovina and Montenegro, is limited to coastal regions. Currently, there are no reports on the evolution of its distribution in the South American continent, suggesting that the unfilling can also partially be due to other factors (e.g. ecological interactions such as competition with native small carnivorans, as for example the South American coati *Nasua nasua* or the greater grison *Galictis vittata*). However, the populations introduced in Europe are currently spreading^11,12^, suggesting the possibility that the species might occupy currently unoccupied favourable spaces (filling the niche), or even spaces that have not been identified as favourable (expansion of the niche), in latter stages of the colonization.

The differences in bioclimatic conditions between the different regions where the species is present demonstrated the necessity of including occurrences from both native and non-native areas in order to model the bioclimatic niche of the small Indian mongoose. Furthermore, the niche conservatism concept is based on the assumption of niche equilibrium, i.e. in the non-native area, the species is present in all the available niches, and absent in all areas not in accordance with its ecological requirements. However, regarding the small Indian mongoose, as the Balkan populations are spreading, we cannot exclude the possibility of niche expansion in later stages of colonization^98^. Hence, the niche of the small Indian mongoose cannot be considered conserved in the different regions of introduction.

### Assessment of the modeled bioclimatic envelop

In this study, we modelled the bioclimatic niche of the small Indian mongoose based on five non-intercorrelated bioclimatic variables, and occurrences from both native and non-native range. In addition, occurrences were filtrated in the environmental space derived from the combination of the five selected bioclimatic variables, and pseudo-absences were randomly generated outside this environmental space occupied by the species. The modelled responses to the bioclimatic variables appear sharper for the mean temperature of the warmest quarter (bio10), the mean diurnal range (bio2) and the precipitation of the coldest quarter (bio19), revealing the preference of the small Indian mongoose for environments with warm summers, low to medium diurnal temperature ranges, and low precipitation in winter. Responses for the temperature annual range (bio7) and the precipitation of the wettest month (bio13) appear less restrictive, showing that the species can cope with environments experiencing high temperature variation throughout the year, and extreme pluviometric conditions during short periods. These results are consistent with prior ecological knowledge of the species, whose distribution cover regions characterized by high temperatures as well as extreme precipitation conditions ranging from the arid Middle East to the humid climate northern India and Myanmar.

### Areas currently favourable and implications for insular ecosystems

Projections reveal highly favourable areas extending broadly within tropical and sub-tropical latitudes, notably encompassing a high number of islands. Insular environments are particularly vulnerable to the introduction of alien species, especially carnivores, which are often poorly represented in native ecosystems^99–101^. The negative impact of introduced small Indian mongooses on insular environments has been significantly documented. The introduction of small Indian mongooses has been correlated with the decline of several amphibian, reptile, bird and mammal species in Hawaii, the Caribbean, the Adriatic, and Japan^9,22^. Moreover, some authors suggested that the small Indian mongoose was implicated in the extirpation and extinction of several species, such as the extirpation of the Black racer *Alsophis ater* in Jamaica^102^, or the Viti Barred Treeskink *Emoia trossula* in the Fiji island of Viti Levu^103^. The negative impact of the small Indian mongoose on insular ecosystems demonstrates the importance to identify and monitor islands where environmental conditions are favourable for the species. Our model identified such conditions in several islands close to regions where the species is already present, in Hawaii, the Caribbean and the Mediterranean regions, and in the Japanese archipelago, but also in the Mascarenes and Oceania. Despite the knowledge of the risks related to the presence of the species in these environments, introductions of individuals are still occurring, as for example in New Caledonia in 2010 (P. Barrière, comm. Pers.^104,105^), or on the Hawaiian island of Kaua’i (Hawaii) in 2012^106^. In both cases, fortunately, the individuals were trapped and no further evidence of the presence of the species was found. However, these few examples may justify increased vigilance in the regions highlighted in our projections, as well as the implementation of surveillance methods and biosecurity protocols to prevent new introductions and to avoid the risk of new colonization. Such biosecurity measures may consist in the awareness of the authorities and agents, the use of tracking tunnels, traps, or poison baits in ports or other appropriate areas, or the use of detection dogs, in addition to the availability of personnel who can act quickly from first detections, as well as general public education programs^106–108^.

### Future favourable areas and implications for spreading populations in Europe

Projections adjusted to bioclimatic conditions by 2050 highlight a significant stability of areas currently favourable to the small Indian mongoose. In addition, lost areas appear to be very limited, and broad new favourable areas extend in the North and South American continents, Africa and Australia, and particularly in Eastern Europe, the Caucasus, and Central Asia. Our climate change projections predicted an expansion of areas favourable to the small Indian mongoose by nearly a quarter of the current area. This expansion contrasts with the predicted northward shift in other meso-carnivores, such as the raccoon, also introduced in the Caribbean, Europe, and Japan^69,109^. This difference arises from the accordance between the bioclimatic characteristics identified favourable to the species by our model, and the consequences of climate change. In southern and eastern Europe, for example, climate change is likely to lead to an overall increase in temperature, an increase in high temperature events (especially during the hottest periods of the year), an increase in extreme precipitation, and a decrease in ice coverage^110,111^, which is a set of climate features that we identified as being favourable to the presence of small Indian mongoose.

Finally, Europe, and more precisely the coastal region of the Balkans, is the only region of introduction of the small Indian mongoose where populations appear to be currently spreading (estimated at 7.5 km per year by Ćirović *et al*.^11^). These populations appear mainly distributed in coastal regions, but Tvrkovic and Krystufek^112^ also reported the presence of mongooses in the Mostar region, several kilometers further inland in Bosnia and Herzegovina. However, some authors suggested that the natural dispersal of small Indian mongooses in the region should be limited by the mountainous areas in the eastern Balkans, characterized by very low temperatures and the retention of snow cover over several days of the year^11,12^. Current projections from our model appear to be in accordance with this hypothesis. However, by 2050, this barrier was predicted to become much more porous, and wider corridors of favourable spaces may appear in the north and in the south of the region, which may offer crossing points to Italy, but also to the newly favourable areas in Eastern Europe, the Caucasus and Central Asia. As the small Indian mongoose is known to impact the populations of various vertebrate species^9,22^, a future potential colonization of these areas could represent a threat for the local biodiversity. The herpetofauna would be particularly at risk in this region which shelters the highest diversity of amphibians and reptiles in Europe^113^. Conversely, interactions with other native meso-predators, such as competition and predation, may mitigate this impact on the continental areas. However, interactions between the small Indian mongoose and the native predator community on the continent remain largely unknown.

## Conclusion

The small Indian mongoose is now recognised as one of the wild carnivore species with the greatest impact on the biodiversity of the introduced regions^32,33^. Our results highlight the tolerance of the species to very high temperature and a wide range of precipitation conditions, resulting in extensive regions currently favourable to the small Indian mongoose within the tropical and sub-tropical latitudes, including a large number of islands. Moreover, predictions for 2050 reveals extensive newly favourable areas, particularly in the Balkan area where the species has been introduced and where the populations are currently spreading. Our results thus provide a new source of useful information for the management of island and continental ecosystems potentially threatened by the spread and potential expansion of the small Indian mongoose under climate change.

## Supplementary information

Supplementary information.

Supplementary information 2.

Supplementary information 3.

Supplementary information 4.

Supplementary information 5.

Supplementary information 6.

Supplementary information 7.

Supplementary information 8.

Supplementary information 9.

Supplementary information 10.

Supplementary information 11.

Supplementary information 12.

## Data Availability

Occurrence data are available in Table S1 of Supplementary Information online. All climate GIS layers are available as raster grids from the Worldclim database: www.worldclim.org.
