# Author Correction: The globally invasive small Indian mongoose *Urva auropunctata* is likely to spread with climate change

**DOI:** 10.1038/s41598-020-68558-2

**Published:** 2020-07-09

**Authors:** Vivien Louppe, Boris Leroy, Anthony Herrel, Géraldine Veron

**Affiliations:** 1 Institut de Systématique, Evolution, Biodiversité (ISYEB), Muséum national d’Histoire naturelle, CNRS, Sorbonne Université, EPHE, Université des Antilles, 57 rue Cuvier, CP 51, 75231 Paris, Cedex 5, France; 2 Unité Biologie des Organismes et Ecosystèmes Aquatiques (BOREA UMR 7208), Muséum National d’Histoire Naturelle, Sorbonne Universités, Université de Caen Normandie, Université des Antilles, CNRS, IRD, Paris, France; 3 Département Adaptations du Vivant (FUNEVOL, UMR 7179), Muséum National d’Histoire Naturelle, CNRS, Paris, France

Correction to: *Scientific Reports* 10.1038/s41598-020-64502-6, published online 04 May 2020

The Acknowledgements section in this Article is incomplete.

“We sincerely thank J.Thevenot (UMS Patrinat), O. Lorvelec (INRA, ESE, Rennes, France), B. Guillemot (Office National de la Chasse et de la Faune Sauvage), H. Magnin and G. Van Laere (Parc National de Guadeloupe), J. Chalifour (Réserve Nationale Naturelle de Saint-Martin), B. Angin (Ardops Environnement), A. Lenoble (PACEA, Université de Bordeaux), M. Rutherford (The University of West Indies, Trinidad), and F. Catzeflis (Institut des Sciences de l’Evolution, Université Montpellier 2, Montpellier, France) for sharing occurrence data. We thank the curators of the museums for providing access to specimens and data (see Table S1).”

Should read:

"We sincerely thank J.Thevenot (UMS Patrinat), O. Lorvelec (INRA, ESE, Rennes, France), B. Guillemot (Office National de la Chasse et de la Faune Sauvage), H. Magnin and G. Van Laere (Parc National de Guadeloupe), J. Chalifour (Réserve Nationale Naturelle de Saint-Martin), B. Angin (Ardops Environnement), A. Lenoble (PACEA, Université de Bordeaux), M. Rutherford (The University of West Indies, Trinidad), and F. Catzeflis (Institut des Sciences de l’Evolution, Université Montpellier 2, Montpellier, France) for sharing occurrence data. We thank the curators of the museums for providing access to specimens and data (see Table S1).

 This project received funding from the region Ile de France (ARDoC) and from the Investissement d’Avenir Project Labex BCDiv (ANR-10-LABX-0003).”

