# Supplementary figures and images for: The globally invasive small Indian mongoose Urva auropunctata is likely to spread with climate change
Source: Sci Rep. 2020 May 4;10:7461. doi: 10.1038/s41598-020-64502-6 (PMC7198557; doi:10.1038/s41598-020-64502-6)

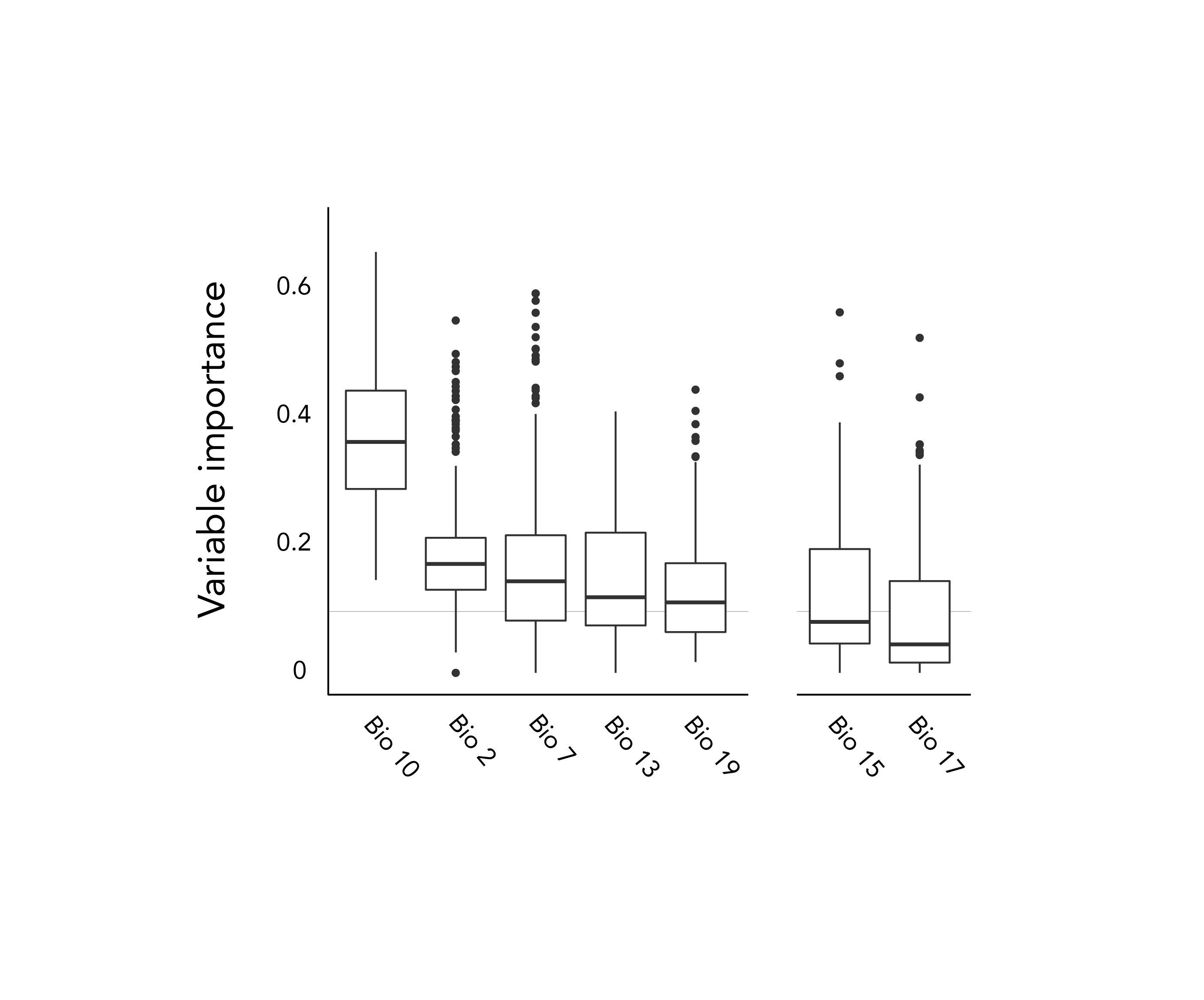

Supplement: Supplementary file 2 — Supplementary information 2. [file 41598_2020_64502_MOESM2_ESM.png]

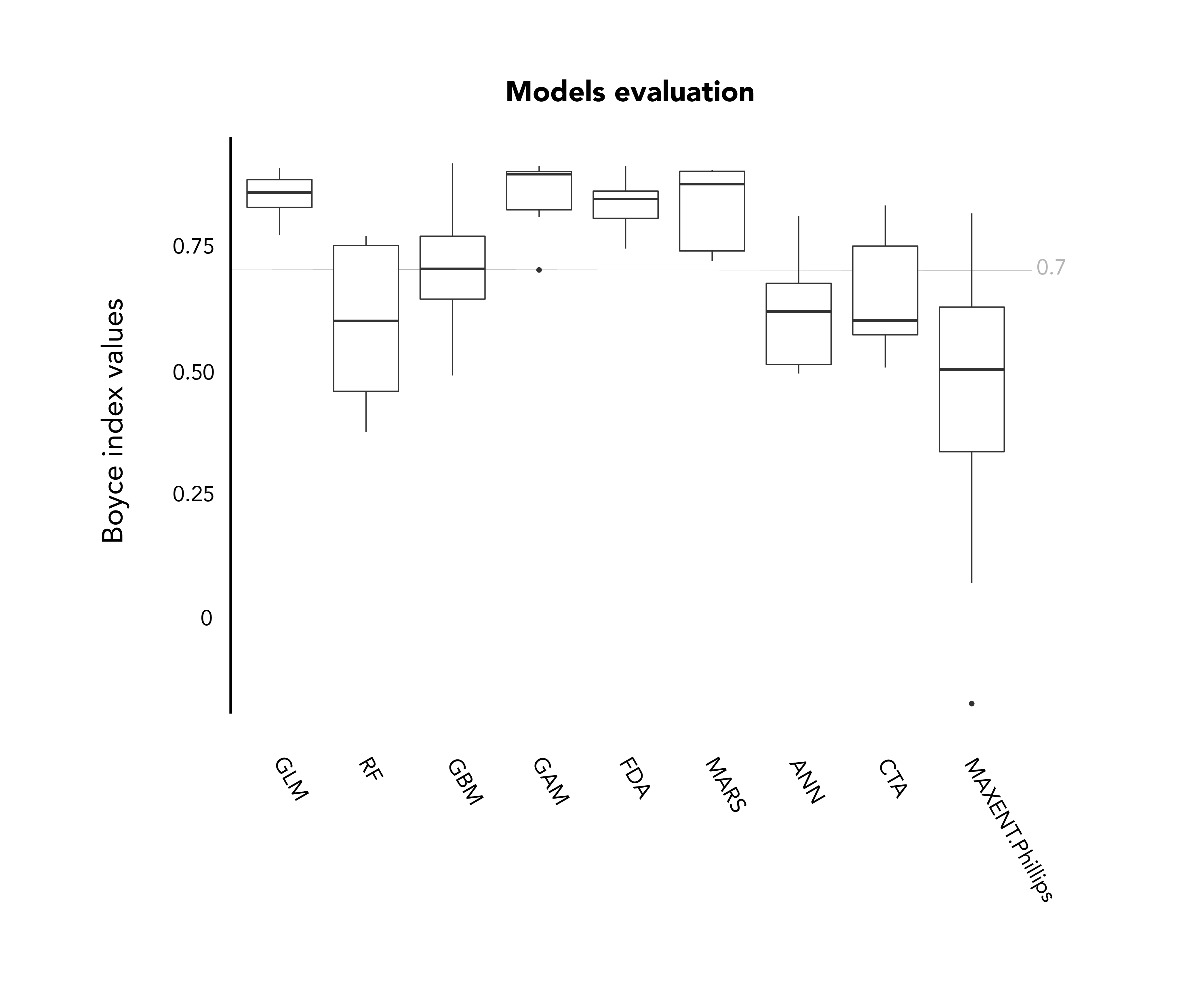

Supplement: Supplementary file 3 — Supplementary information 3. [file 41598_2020_64502_MOESM3_ESM.png]
