# Supplementary material for: The globally invasive small Indian mongoose Urva auropunctata is likely to spread with climate change
Source: Sci Rep. 2020 May 4;10:7461. doi: 10.1038/s41598-020-64502-6 (PMC7198557; doi:10.1038/s41598-020-64502-6)
Supplement: Supplementary file 7 — Supplementary information 7. [file 41598_2020_64502_MOESM7_ESM.docx]

**Protocol of selection of variables significantly predicting the distribution of *Urva auropunctata*.**

We defined a protocol to identify variables that were not intercorrelated and significantly predicted the suitable range of environmental conditions for the species. This protocol is divided into three stages: first, we identified groups of intercorrelated variables on the basis of a hierarchical ascendant classification with a distance metric based on Pearson’s correlation coefficient. Secondly, we identified the variables best predicting the distribution of the considered species for each group of intercorrelated variables. Thirdly, out of the variables saved at stage 2 and the variables not correlated to any other, we kept the variables that best predicted the distribution of *Urva auropunctata*.

Because our modelling protocol is based on different modelling techniques with various criteria, the predictive power of each variable is difficult to assess and compare between models. Therefore, we applied a method that allows the importance of each variable to be assessed independently of the modelling technique. This method is implemented in BIOMOD as the « variable importance » procedure. The procedure for assessing the importance of a variable for a model is as follows: first, the model is calibrated with all the variables. Secondly, a standard prediction is made on the basis of all the variables. Thirdly, the assessed variable is randomized, and a new prediction is made with the randomized variable. Finally, the correlation between the standard prediction and the prediction with the randomized variable is calculated. If the correlation is high, then the considered variable contributes poorly to the prediction; then the predictive power of the variable is low. Conversely, if the correlation is low, then the predictive power of the variable is high. For each variable, this randomization procedure is computed 10 times. Hence, this procedure provides the average predictive importance of each variable for each of the computed models.

*Step 1. Identification of groups of intercorrelated variables*

We first calculated the Pearson’s correlation coefficients between the 19 bioclimatic variables across the entire world. Then, we calculated the following distance metric among variables: d = (1-r) where r was Pearson’s correlation coefficient. On the basis of these distances, we constructed a hierarchical ascendant classification within which we identified groups of variables that were intercorrelated at a threshold of 0.7 (i.e. distance<0.3). Seven groups of intercorrelated variables were identified: (1) bio15, (2) bio2; (3) bio8, bio5 and bio10; (4) bio9, bio1, bio6, bio11, bio3, bio4 and bio7; (5) bio18, bio12, bio13, and bio16; (6) bio19; (7) bio14 and bio17.

*Step 2. Reduction of variables in groups of intercorrelated variables*

For each group of intercorrelated variables, we calibrated all the models of our modelling protocol. We made 3 pseudo-absence runs with 403 pseudo-absences selected for each run (number equal to the presences). For each run, the variable importance procedure was computed, and we selected the variable with the highest rank among all the models.

*Step 3. Final selection of variables*

Finally, we calibrated all the models with the variables selected at step 2 and the variables that were not correlated to any other, using the same protocol as in step 2. We then selected the variables presenting importance strictly superior to 0.10 for at least 50% of the models.
