# Supplementary material for: The globally invasive small Indian mongoose Urva auropunctata is likely to spread with climate change
Source: Sci Rep. 2020 May 4;10:7461. doi: 10.1038/s41598-020-64502-6 (PMC7198557; doi:10.1038/s41598-020-64502-6)
Supplement: Supplementary file 8 — Supplementary information 8. [file 41598_2020_64502_MOESM8_ESM.docx]

Analysis of niche conservatism

Following the methodology proposed byWarren, Glor and Turelli ^1^⁠, and further developed by Broennimann et al. ^2^⁠, we calculated niche overlap, equivalence, and similarity between the native range (226 occurrences) and the invaded range (177 occurrences).
Niche overlap was calculated using a PCA approach and calibrated with the five selected environmental variables for each area of interest. PCA scores of the species occurrences on the first two axes were projected onto a grid of cells delineated by the minimum and maximum PCA scores of the environmental variables. Next, a kernel density function was applied to estimate a density of occurrence for each cell of the grid (see Di Cola et al. ^3^⁠for methodological details). Thereafter, niche overlap was estimated using Schoener’s D ^4^⁠. This index varies between 0 and 1; 0 meaning no overlap, while 1 meaning identical niches. This index was then used to assess niche equivalence and similarity.
Occurrences in each compared range were pooled and randomly split into two datasets with equal size as the original dataset. Niche overlap was then estimated with the D index. This procedure was repeated 100 times to create a null distribution. The observed D was then compared to these simulated values, and the null hypothesis of equivalence between niches was rejected when observed values fell outside of the 95% confidence interval of the simulated distribution.
Alternatively, niche similarity tests investigate whether the niches in the native or invaded range predict one another better than expected by chance. Occurrences in one area were randomly reallocated within their respective available environmental space and simulated niches were compared to the niche of the other area with both indices. This procedure was repeated 100 times to create a null distribution. The observed D was then compared with the simulated values, and the null hypothesis of similarity of the tested niche to the other was rejected when observed values fell outside of the 95% confidence interval of the simulated distribution.

Along with the niche overlap test, niche expansion and unfilling were also calculated. The expansion index corresponds to environmental conditions in the invaded area that are absent in the native area. Conversely, unfilling refers to environmental conditions in the native area that are absent in the invaded area. Both indices vary between 0 and 1.

All niche conservatism tests were performed using the package ecospat v2.1.1 ^5^ implemented in the R software^6^⁠.

References

1. Warren, D. L., Glor, R. E. & Turelli, M. Environmental niche equivalency versus conservatism: Quantitative approaches to niche evolution. *Evolution (N. Y).* **62**, 2868–2883 (2008).

2. Broennimann, O. *et al.* Measuring ecological niche overlap from occurrence and spatial environmental data. *Glob. Ecol. Biogeogr.* **21**, 481–497 (2012).

3. Di Cola, V. *et al.* ecospat: an R package to support spatial analyses and modeling of species niches and distributions. *Ecography (Cop.).* **40**, 774–787 (2017).

4. Schoener, T. W. The Anolis lizards of Bimini: resource partitioning in a complex fauna. *Ecology* **49**, 704–726 (1968).

5. Broennimann, O. *et al.* Package ‘ ecospat ’. (2016).

6. R Development Core Team. R: A Language and Environment for Statistical Computing. *R Found. Stat. Comput.* (2019).
