# Supplementary material for: The globally invasive small Indian mongoose Urva auropunctata is likely to spread with climate change
Source: Sci Rep. 2020 May 4;10:7461. doi: 10.1038/s41598-020-64502-6 (PMC7198557; doi:10.1038/s41598-020-64502-6)
Supplement: Supplementary file 9 — Supplementary information 9. [file 41598_2020_64502_MOESM9_ESM.docx]

Data preparation and pseudo-absence selection

We followed the methodology developed and assessed by Louppe et al ^1^⁠. The preparation of presence-only data for ENMs included two important steps. The first one consisted of filtering the presence data to reduce autocorrelation and sampling bias (e.g. refs ^2,3^⁠). The second one consisted of selecting pseudo-absence data to calibrate models. Our environmental filtering focuses on the distribution of data in the space of environmental variables. Varela et al^2^⁠ and Louppe et al^1^⁠ showed that removing duplicated records in the environmental space consistently improved the quality of model predictions, contrary to filtering in a geographical space. Therefore, we created a gridded environmental space on the basis of the five selected environmental variables in which we projected all the conditions existing in the geographical space. Next, we projected within this environmental space all the presence points of the species and removed all duplicate points per cell, which resulted in a total of 228 occurrences. To select pseudo-absences within the environmental space, we applied a procedure to avoid selecting pseudo-absences within environmental conditions that are favourable for the species. To do so, we calculated the restricted n-dimensional convex hull of presences, defined as the smallest convex hull encompassing all occurrence points. This restricted convex hull is considered as a proxy of the favourable environmental conditions outside which we randomly generated 228 pseudo-absences (i.e., in equal number to the filtered occurrences) with three repetitions. Such a procedure is supported by the statistical theory of model-based designs, also known as “D-designs” which are assumed to minimize prediction variance ^4^⁠. Overall, the environmental filtering of presences will result in a decreased autocorrelation ^2^⁠ and the convex hull will minimize the risk to sample pseudo-absences inside favourable conditions. In addition, we consider this approach to produce a better description potential distribution of the species compared to geographical filtering approaches, as it corrects for biases linked to heterogeneity in sampling intensity and reduces the risks of generating pseudo-absences falling within favourable areas ^1^⁠. Environmental filtering of presences and selection of pseudo-absences were realized using the R package geometry v0.4.3 ^5^⁠.

References

1. Louppe, V., Leroy, B., Herrel, A. & Veron, G. Current and future climatic regions favourable for a globally introduced wild carnivore, the raccoon Procyon lotor. *Sci. Rep.* **9**, 1–13 (2019).

2. Varela, S., Anderson, R. P., García-Valdés, R. & Fernández-González, F. Environmental filters reduce the effects of sampling bias and improve predictions of ecological niche models. *Ecography (Cop.).* **37**, 1084–1091 (2014).

3. Aiello-Lammens, M. E., Boria, R. A., Radosavljevic, A., Vilela, B. & Anderson, R. P. spThin: An R package for spatial thinning of species occurrence records for use in ecological niche models. *Ecography (Cop.).* **38**, 541–545 (2015).

4. Hengl, T., Sierdsema, H., Radović, A. & Dilo, A. Spatial prediction of species’ distributions from occurrence-only records: combining point pattern analysis, ENFA and regression-kriging. *Ecol. Modell.* **220**, 3499–3511 (2009).

5. Roussel, J.-R. *et al.* Package ‘geometry’. (2019).
