# Supplementary material for: The globally invasive small Indian mongoose Urva auropunctata is likely to spread with climate change
Source: Sci Rep. 2020 May 4;10:7461. doi: 10.1038/s41598-020-64502-6 (PMC7198557; doi:10.1038/s41598-020-64502-6)
Supplement: Supplementary file 10 — Supplementary information 10. [file 41598_2020_64502_MOESM10_ESM.docx]

**Niche similarity and equivalency tests between the niches of the native range and the regions invaded by *Urva auropunctata*.**

Niche conservatism tests show contrasting results.
Niche similarity (similarity of the invaded areas niche to the native niche) was rejected as D indices fell outside the 95% confidence interval of simulated values (*P* = 0.06; Fig. 1), suggesting that the niche in invaded areas is not more similar to the native niche than expected if the distribution in the invaded areas was random.
Conversely, niche equivalence was not rejected (*P* < 0.05; Fig. 1), suggesting that both invaded areas and native niches are more comparable than expected with a random distribution over both areas.
