# Supplementary material for: The globally invasive small Indian mongoose Urva auropunctata is likely to spread with climate change
Source: Sci Rep. 2020 May 4;10:7461. doi: 10.1038/s41598-020-64502-6 (PMC7198557; doi:10.1038/s41598-020-64502-6)
Supplement: Supplementary file 12 — Supplementary information 12. [file 41598_2020_64502_MOESM12_ESM.docx]

**Supplementary information**

Table S1

Occurrence data and sources or references.
Museum acronymes: AMNH: American Museum of Natural History (New York, USA); BNHS: Bombay Natural History Society Museum (Mumbai, India); BPBM: Bishop Museum Natural History (Honolulu, USA); BZM: Museum fur Naturkunde/Zoologische Museum der Humboldt-Universitat (Berlin, Germany); CAS: California Academy of Science (San Francisco, USA); FLMNH: Florida Museum of Natural History (Gainesville, USA); FS: Forschungsinstitut Senckenberg (Frankfurt/Main, Germany); HALLE: Zoological Collection of the Institute of Zoology, Martin-Luther-Universitat Halle-Wittenberg (Halle, Germany); HZM: Harrison Institute (Sevenoaks, UK); MCZ: Harvard Museum of Comparative Zoology (Cambridge, USA); MHNGr: Muséum d'Histoire Naturelle de Grenoble (Grenoble, France); MNHN: Museum National d'Histoire Naturelle (Paris, France); MRAC: Musée Royal de l'Afrique Centrale (Tervuren, Belgium); MV: Museum Victoria (Melbourne, Australia); MZLU: Lund Museum of Zoology (Lund, Sweden); NHM: Natural History Museum (Londres, UK); PSM: Slater Museum, University of Puget Sound (Takoma, USA); ROM: Royal Ontario Museum (Toronto, Canada); SMNS: Staatliches Museum fur Naturkunde (Stuttgart, Germany); USNM: National Museum of Natural History (Washington, USA); ZFMK: Zoologisches Forschungsmuseum Alexander Koenig (Bonn, USA); ZSI: Zoological Survey of India (Calcutta, india).

Figure S1

Groups of intercorrelated environmental variables.

Figure S2

Importance of the environmental variables used in modelling.

Figure S3

Boyce index values.

Figure S4

Projection of standard deviation values of global bioclimatic favourability for the small Indian mongoose. Figure generated using the softwares R v3.5 ([https://www.r-project.org](https://www.r-project.org/)) and QGIS v2.14.20 (https://qgis.org/).

Figure S5

Projection of current global bioclimatic favourable for the small Indian mongoose.
Unfavourable: areas that are currently unfavourable remain unfavourable in the future; Favourable: areas that are currently favourable. Orange circles represent occurrences used in the modelling. Figure generated using the softwares R v3.5 ([https://www.r-project.org](https://www.r-project.org/)) and QGIS v2.14.20 (https://qgis.org/).

Figure S6

Predicted favourable range change for the small Indian mongoose by 2050 according to scenario RCP2.6. Unfavourable: areas that are currently unfavourable and are predicted to remain unfavourable in the future; Become unfavourable: areas currently favourable that will become unfavourable nature in the future; Maintained favourable: areas that are currently favourable and will remain favourable in the future; Newly favourable: areas that are currently not favourable but would become favourable in the future. Figure generated using the softwares R v3.5 ([https://www.r-project.org](https://www.r-project.org/)) and QGIS v2.14.20 (https://qgis.org/).

Method S1

Protocol of selection of variables significantly predicting the distribution of the small Indian mongoose.

Method S2

Protocol for the analysis of niche conservatism of the small Indian mongoose.

Method S3

Protocol for data preparation and pseudo-absence selection.

Results S1

Niche similarity and equivalency tests between the niches of the native range and the regions invaded by the small Indian mongoose *Urva auropunctata*.
